# Physician Experiences With and Perspectives on Clozapine Prescribing

**DOI:** 10.1001/jamanetworkopen.2024.59311

**Published:** 2025-02-13

**Authors:** Ameet Sarpatwari, Zhigang Lu, Massimiliano Russo, Heidi Zakoul, Su Been Lee, Gita A. Toyserkani, Esther H. Zhou, Cynthia LaCivita, Katherine Hyatt Hawkins Shaw, Gerald J. Dal Pan, Aaron S. Kesselheim

**Affiliations:** 1 Program on Regulation, Therapeutics, and Law, Division of Pharmacoepidemiology and Pharmacoeconomics, Department of Medicine, Brigham and Women’s Hospital, Boston, Massachusetts; 2 Harvard Medical School, Boston, Massachusetts; 3 Department of Statistics, The Ohio State University, Columbus; 4 Division of Pharmacoepidemiology and Pharmacoeconomics, Department of Medicine, Brigham and Women’s Hospital, Boston, Massachusetts; 5 Office of Surveillance and Epidemiology, Center for Drug Evaluation and Research, US Food and Drug Administration, Silver Spring, Maryland

## Abstract

**Importance:**

Clozapine is an effective medication for treatment-resistant schizophrenia but can cause agranulocytosis. Since its approval, patients taking clozapine have been required to undergo regular blood testing to screen for neutropenia. In 2015, the risk management programs were merged into a single risk evaluation and mitigation strategy (REMS), which mandates that prescribers undergo a certification process in which they attest to understanding the risk of agranulocytosis and commit to performing required blood testing. It is unknown how the clozapine REMS program has affected clinical practice.

**Objective:**

To understand physicians’ perceptions of and experiences with the clozapine REMS program.

**Design, Setting, and Participants:**

This survey study included US physicians who prescribed clozapine in 2021 or 2022. A 57-question, closed-field questionnaire was administered via paper or online. The first survey wave took place from May to October 2022 and the second from October 2022 to January 2023.

**Exposures:**

Gender, race and ethnicity, practice specialty, practice setting, practice region, professional time in clinical practice, years since graduation from medical school, and number of patients prescribed clozapine in the past 3 years.

**Main Outcomes and Measures:**

Experiences with and perceptions of the REMS certification process, patient initiation, and the REMS safe use requirements were measured using Likert scales. Descriptive statistics and multivariable modeling were used to analyze the study results.

**Results:**

Of 750 physicians contacted, 196 responded (30% response rate). Most respondents were male (129 [67%]), most identified as Asian (48 [25%]) or White (124 [63%]), and most were psychiatrists (165 [86%]); almost half (88 [45%]) practiced in an outpatient group setting. Most respondents reported that the information provided during certification was clear (179 [92%]) and contained useful drug information (145 [75%]) and that the paperwork associated with required blood testing facilitated physician-patient conversation (102 [53%]). Most respondents wanted certification materials to include all clinically important risks (172 [89%]) and benefits (139 [72%]) and reported frequent delays in medication access owing to required blood testing (115 [60%]). Two-thirds of respondents agreed that the positives of the certification process (123 [64%]) and blood testing requirements (129 [68%]) outweighed the negatives. In multivariable analysis, negative REMS perceptions were less common among physicians in practice longer (≥35 vs <15 years since medical school: odds ratio [OR], 0.28; 95% CI, 0.10-0.80) and among American Indian or Alaska Native, Black, and Native Hawaiian or Other Pacific Islander physicians compared with White physicians (OR, 0.08; 95% CI, 0.01-0.73).

**Conclusions and Relevance:**

In this survey study of physician prescribers of clozapine, a majority were satisfied with the clozapine REMS program but highlighted the need for enhanced educational materials and administrative efficiency.

## Key Points

**Question:**

How do physician prescribers of clozapine perceive its risk evaluation and mitigation strategy (REMS) program?

**Findings:**

In this survey study of 196 physicians, two-thirds of respondents reported that the program’s positives outweighed its negatives, with negative perceptions more common among White respondents and more recent medical school graduates. Most physicians reported that information provided under the program was clear and that paperwork associated with required blood testing facilitated conversation, but they also noted delays in medication access and a desire for information on benefits and non-REMS–related risks.

**Meaning:**

These findings suggest that most physicians surveyed were satisfied with the clozapine REMS program, but they indicated its educational materials and administrative efficiency can be enhanced.

## Teaser

This survey study examines physician-prescriber experiences with and perspectives on the clozapine risk evaluation and mitigation strategy.

## Introduction

In 1989, the US Food and Drug Administration (FDA) approved clozapine (Clozaril; Novartis) for treatment-resistant schizophrenia.^1^ Preapproval testing demonstrated clozapine’s marked efficacy,^2,3^ and subsequent observational studies confirmed its marked effectiveness.^4,5^ Yet clozapine use has been modest in the US. Although an estimated 30% of people with schizophrenia have the treatment-resistant form,^6,7^ by the late 2000s, fewer than 10% of people with schizophrenia were treated with clozapine in 44 states.^8^

A key reason for low clozapine use has been its perceived safety, specifically concern over its risk of agranulocytosis (absolute neutrophil count <500/μL of blood [to convert to ×10^9^/L, multiply by 0.001]). Shortly after clozapine launched in Finland in 1979, 18 of 2000 patients (0.9%) taking it developed agranulocytosis, resulting in 8 deaths.^9^ To secure FDA approval, clozapine’s manufacturer developed the Clozaril Patient Management System, in which patients had to obtain a white blood cell count (WBC) and a blood differential prior to initiation and to undergo weekly blood testing (WBC count monitoring) while receiving treatment and for 4 weeks following treatment.^10^

In September 2015, under the authority granted by the FDA Amendments Act of 2007,^11^ the FDA approved a new centralized monitoring program for all manufacturers called the clozapine risk evaluation and mitigation strategy (REMS), which included the need for prescriber certification in addition to a safe use requirement (blood testing). As part of the certification process, prescribers received educational materials about the risk of agranulocytosis and the requirements of the clozapine REMS program, completed a knowledge assessment, and were asked to attest on the prescriber enrollment form that they had reviewed the educational materials and successfully completed the knowledge assessment. In 2021, the REMS was modified and required prescribers to submit monthly patient status forms, pharmacies had to verify the safe use condition via a website or call center, and prescribers and pharmacies had to complete a one-time recertification.^12^

The clozapine REMS program has long been controversial.^13^ Because the occurrence of agranulocytosis is infrequent (1.3% cumulative incidence at 1 year),^14^ some physicians, patients, and caregivers have expressed concern that the requirements to mitigate this risk have resulted in fewer would-be prescribers and gaps in treatment, with harmful consequences for patients.^13,15^ The 2021 REMS modification also included a change in the vendor that linked to a new system to support the clozapine REMS program, which required prescribers and pharmacists to recertify. The need to recertify, coupled with high call volume and long wait times, frustrated prescribers and patients,^16^ prompting the FDA to temporarily permit dispensing to patients outside the REMS program.^17^ To understand how US physicians perceive that the clozapine REMS program has affected their clinical practice, we conducted a national survey of prescribers.

## Methods

The Mass General Brigham institutional review board approved this survey study. Participants provided informed consent. The study followed the American Association for Public Opinion Research (AAPOR) Best Practices for Survey Research.

### Survey Instrument Development

We created and tested a survey instrument with 57 closed-field questions (eAppendix in Supplement 1). These questions used 5-point Likert scales and were arranged around 3 topics: (1) the prescriber certification process, (2) the process for initiating clozapine treatment for a patient, and (3) the safe use requirement. We also solicited demographic information, which we believed might affect physician perceptions and experiences, including gender, race and ethnicity, clinical specialty, practice setting, years since medical school, and prescribing frequency. Participants self-reported ethnicity (Hispanic) and race (American Indian or Alaska Native, Asian, Black, Native Hawaiian or Other Pacific Islander, or White). We pretested the survey with a physician prescriber of clozapine and refined wording based on the cognitive interview.

### Survey Recruitment and Administration

Luminas, a survey research firm, administered the survey. From IQVIA, we purchased a first sample of 500 randomly drawn US-based physicians recorded as having prescribed clozapine in 2021 or 2022. For each physician, we made up to 4 postal contacts and 4 email contacts. Postal contacts included a cover letter, an information sheet, a paper survey, and a postage-paid return envelope. The first postal contact also included a $20 check. The cover letter and email contacts included a unique URL for online completion. Physicians who completed a survey could claim an $80 e-gift card. A second sample of 250 physicians was subsequently purchased from IQVIA using the same inclusion criteria, and a second period of field testing was employed. The first wave took place from May to October 2022, and the second from October 2022 to January 2023.

### Statistical Analysis

The response rate was calculated using the AAPOR third standard definition.^18^ Responses from partially completed surveys were included, with descriptive statistics for each question calculated based on the number of received responses.

Multivariable logistic regression modeling was used to examine predictors of physicians dissatisfied with the clozapine REMS program. For this model, a negative perception variable was constructed based on “somewhat disagree” or “strongly disagree” responses to either of 2 statements: “Overall, the positives of the provider certification process for clozapine outweigh the negatives” or “Overall, the patient safe use requirements for clozapine outweigh the negatives.” Covariates in the model included gender, race, practice specialty, practice setting, practice region, professional time in clinical practice, years since graduation from medical school, and number of patients prescribed clozapine in the past 3 years. In univariate and multivariate models, race was collapsed into 3 groups: Asian, White, and race other than Asian and White, which included American Indian or Alaska Native, Black, and Native Hawaiian or Other Pacific Islander physicians.

*P* < .05 (2-tailed) was considered statistically significant. Analyses were performed using SAS, version 9.4 (SAS Institute).

## Results

Of 750 physicians contacted, 196 returned a partially or fully completed survey (30% response rate). Most respondents were male (129 [67%]), most were psychiatrists (165 [86%]), and almost half (88 [45%]) practiced in an outpatient group setting (Table). Participants identified as American Indian or Alaska Native (1 [0.5%]), Asian (48 [25%]), Black (8 [4%]), Hispanic (6 [3%]), Native Hawaiian or Other Pacific Islander (1 [0.5%]), or White (124 [63%]); 15 (8%) preferred not to answer.

**Table.** Participant Demographics

Characteristic	No. (%) (N = 196)^a^
Gender	
Male	129 (67)
Female	59 (31)
Prefer not to answer	5 (3)
Race	
American Indian or Alaska Native	1 (0.5)
Asian	48 (25)
Black	8 (4)
Native Hawaiian or Other Pacific Islander	1 (0.5)
White	124 (63)
Prefer not to answer	15 (8)
Ethnicity	
Hispanic	6 (3)
Time since medical school graduation, y	
<15	31 (16)
15-24	33 (17)
25-34	38 (20)
≥35	92 (47)
Specialty	
Psychiatry	165 (86)
Internal medicine	18 (9)
Neurology	7 (4)
Geriatrics	2 (1)
In what clinical settings do you prescribe clozapine? (multiple answers permitted)	
Outpatient clinic (solo practice)	35 (14)
Outpatient (group practice)	88 (45)
Community hospital (nonmilitary or VA)	33 (14)
Academic hospital (nonmilitary or VA)	28 (12)
Military or VA hospital	1 (0.4)
Other	58 (24)
What percentage of your professional time is spent in direct patient care?	
<80	38 (20)
80-89	46 (24)
90-99	65 (33)
100	45 (23)
No. of patients to whom clozapine was prescribed in past 3 y	
1-10	90 (46)
11-20	41 (21)
≥21	64 (33)
US region	
Northeast	68 (35)
South	39 (20)
Midwest	55 (28)
West	34 (17)

Abbreviations: VA, US Department of Veterans Affairs.

^a^ Percentages were based on number of responses to each question.

### Perceptions of Clozapine Risk

The majority of respondents (115 [64%]; 95% CI, 57%-71%) ranked severe neutropenia as the biggest concern among the following 4 drug risks: (1) severe neutropenia; (2) orthostatic hypotension, bradycardia, or syncope; (3) pulmonary embolism; and (4) seizure. One-fourth of physicians (43 [24%]; 95% CI, 18%-30%) ranked orthostatic hypotension, bradycardia, or syncope first. By contrast, fewer respondents ranked pulmonary embolism (11 [6%]; 95% CI, 3%-9%) or seizure (10 [6%]; 95% CI, 2%-10%) first. In learning about the risks of clozapine, respondents found clinical decision support tools (57 [33%]; 95% CI, 26%-40%), the drug’s labeling (50 [29%]; 95% CI, 22%-36%), and medical journal articles (46 [26%]; 95% CI, 20%-32%) most useful.

### Prescriber Certification

About two-thirds of respondents (123 [64%]; 95% CI, 57%-71%) agreed that the positives of the certification process outweighed the negatives. A similar proportion (128 [66%]; 95% CI, 59%-73%) agreed it was reasonable that certification was required to prescribe clozapine and not for other drugs they used to treat schizophrenia; half (99 [51%]; 95% CI, 44%-58%) found completing the certification process easy (eFigure 1 in Supplement 1). Almost all respondents (179 [92%]; 95% CI, 88%-96%) agreed that the certification process clearly explained the testing required of patients taking clozapine; three-fourths (145 [75%]; 95% CI, 69%-81%) agreed it provided useful information about the drug, including the risk of severe neutropenia (190 [97%]; 95% CI, 95%-99%).

When asked how the certification process could be improved, almost nine-tenths of respondents (172 [89%]; 95% CI, 85%-93%) agreed that educational materials should include information on all clinically important risks of clozapine; three-fourths (139 [72%]; 95% CI, 66%-78%) agreed on inclusion of a summary of how well clozapine is expected to work. Fewer respondents (116 [60%]; 95% CI, 53%-67%) agreed that certification should include a quiz covering knowledge of clozapine risks and required testing, and only one-fourth (46 [24%]; 95% CI, 18%-30%) agreed that prescribers should need to undergo the certification process annually (as opposed to once under the clozapine REMS program).

### Blood Testing Requirement

As with the certification process, two-thirds of respondents (129 [68%]; 95% CI, 61%-75%) agreed that the positives of the safe use requirement for clozapine (ie, blood testing) outweighed the negatives. Three-fourths of respondents (150 [77%]; 95% CI, 71%-83%) agreed that the safe use requirement was clinically necessary, and half (102 [53%]; 95% CI, 46%-60%) agreed that paperwork associated with the safe use requirement facilitated discussion about clozapine with patients (eFigure 2 in Supplement 1). Almost all respondents reported discussing the risk of agranulocytosis with patients always or almost always (171 [88%]; 95% CI, 83%-93%) or most of the time (9 [5%]; 95% CI, 2%-8%). More than half of respondents (118 [61%]; 95% CI, 54%-68%) reported that their patients always or almost always adhered to the blood testing schedule set forth in the REMS program, and more than one-quarter (56 [29%]; 95% CI, 23%-35%) did most of the time.

However, almost half of respondents (85 [44%]; 95% CI, 37%-51%) reported that testing under the clozapine REMS program was somewhat or very hard. Majorities agreed that the safe use requirement was burdensome for patients (135 [71%]; 95% CI, 65%-77%) and often caused a delay in patients receiving clozapine (115 [60%]; 95% CI, 53%-67%).

### Univariate Analysis

**Figure 1.**
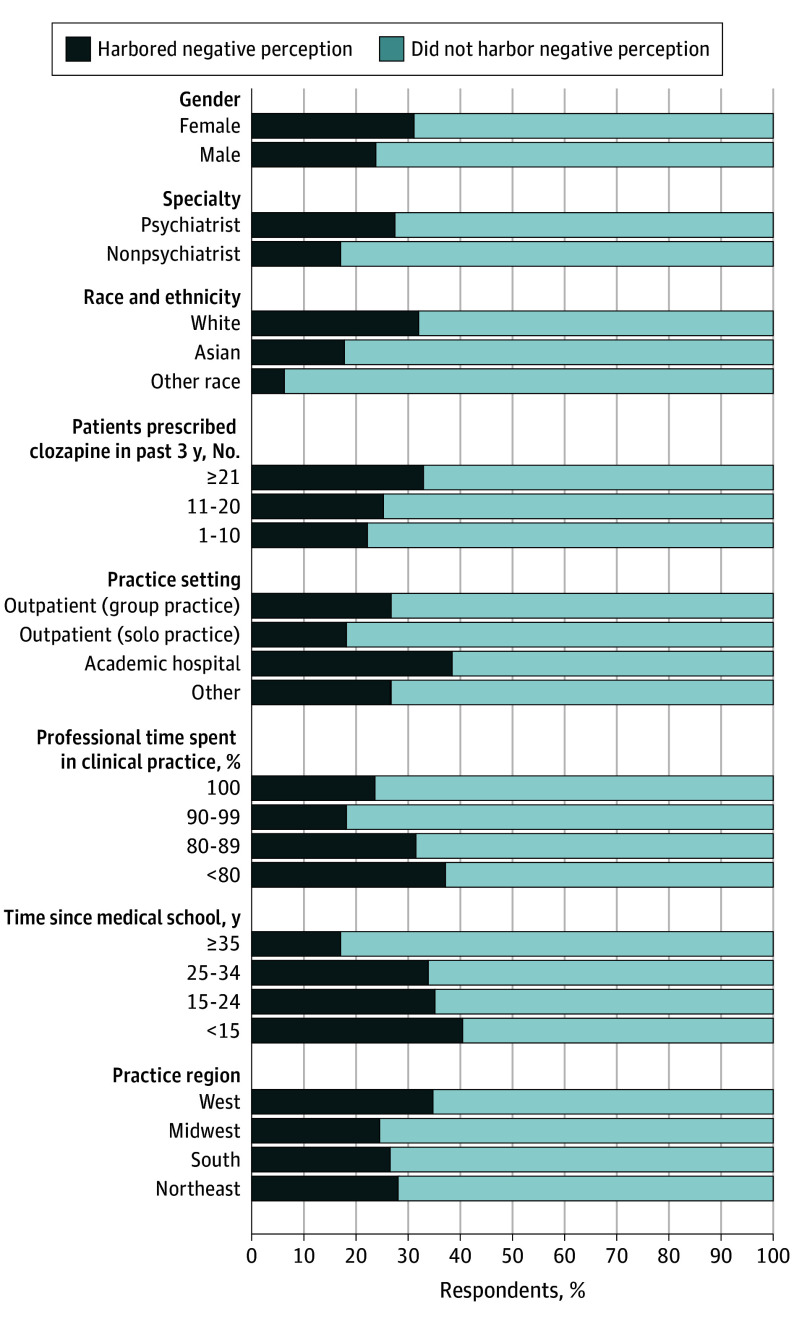
Negative Clozapine Risk Evaluation and Mitigation Strategy (REMS) Perception Among Respondents Answering Each Contributing Question to the Multivariable Model Harboring a negative perception was defined as answering “somewhat disagree” or “strongly disagree” to either of 2 questions: “Overall, the positives of the provider certification process for clozapine outweigh the negatives” or “Overall, the patient safe use requirements for clozapine outweigh the negatives.” Other race includes American Indian or Alaska Native, Black, or Native Hawaiian or Other Pacific Islander.

The multivariable model included 176 respondents who answered each contributing question. Within this cohort, a negative perception of the clozapine REMS program was more common among White respondents (38 [33%]; 95% CI, 24%-42%) than among American Indian or Alaska Native, Black, Native Hawaiian of Other Pacific Islander respondents (1 [6%]; 95% CI, 0%-17%) (*P* = .11) (Figure 1). A greater proportion of respondents in practice for a shorter period (<15 years; 12 [43%]; 95% CI, 25%-61%) also had a negative perception of the clozapine REMS program compared with those in practice for 15 to 24 years (11 [34%]; 95% CI, 18%-50%), 25 to 34 years (12 [34%]; 95% CI, 18%-50%), or 35 years or more (15 [19%]; 95% CI, 10%-28%) (*P* = .048).

### Multivariable Analysis

**Figure 2.**
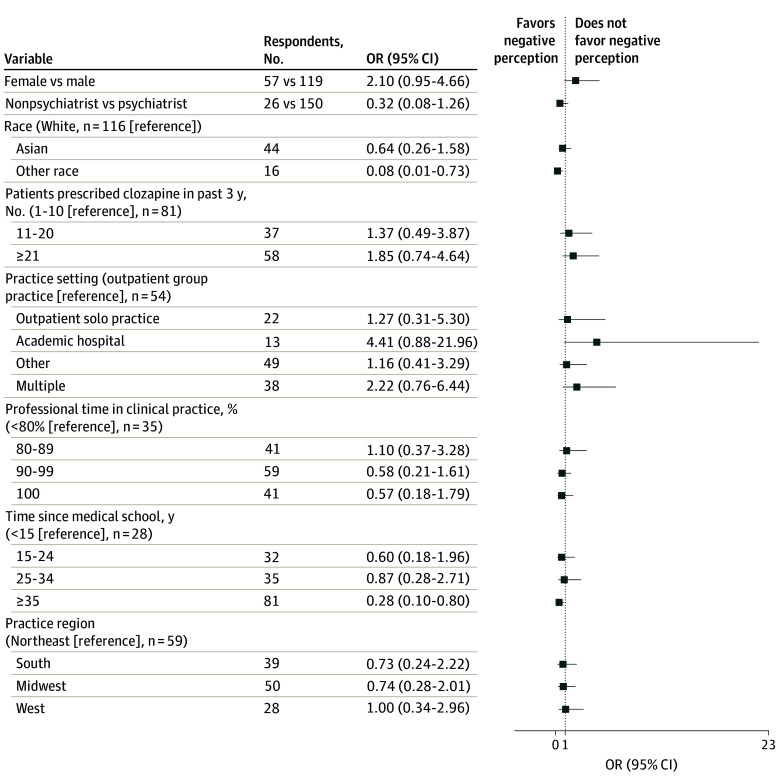
Association Between Demographic Variables and a Negative Perception of the Clozapine Risk Evaluation and Mitigation Strategy (REMS) A negative perception was defined as answering “somewhat disagree” or “strongly disagree” to either of 2 questions: “Overall, the positives of the provider certification process for clozapine outweigh the negatives” or “Overall, the patient safe use requirements for clozapine outweigh the negatives.” Other race includes American Indian or Alaska Native, Black, or Native Hawaiian or Other Pacific Islander. OR indicates odds ratio.

In the multivariable model, a negative perception of the clozapine REMS program was associated with 2 variables: years since medical school and race (Figure 2). Respondents who had been in practice longer were less likely to have a negative view (≥35 vs <15 years since medical school: odds ratio [OR], 0.28; 95% CI, 0.10-0.80; *P* = .02), as were non-Asian and non-White respondents compared to White respondents (OR, 0.08; 95% CI, 0.01-0.73; *P* = .03).

## Discussion

In this national survey, two-thirds of physician prescribers of clozapine who responded agreed that the positive features of the clozapine REMS program outweighed the negatives despite controversy over its need and impact. Underlying this perspective, a majority of physicians ranked neutropenia as the foremost clozapine risk, whereas a larger proportion identified positive features of each REMS component. These features included the useful drug information and clear explanation of testing requirements provided as part of the certification process and the physician-patient discussion fostered by the blood testing requirement. Survey responses also highlighted potential changes to the clozapine REMS program, including (1) adding clinically important, non-REMS–related drug risks and drug effectiveness data in certification materials and (2) addressing the challenge of blood testing to reduce possible interruptions in treatment.

The generally positive perception of the clozapine REMS program that the survey uncovered contrasts with the criticism it has received from some academic physicians and professional societies for hindering access to a critical, effective medication. The diversity of respondents as to practice setting, practice region, professional time in clinical practice, years since graduation from medical school, and frequency of prescribing supports the broad generalizability of the survey findings. This suggests that strong opposition to the clozapine REMS program may not be widespread or may be limited to the delays that the program may cause.

Multivariable modeling revealed associations of practice type and years since medical school with a negative perception of the clozapine REMS program, with greater dissatisfaction seen among physicians practicing in academic hospitals and in practice fewer years. One hypothesis for this finding is that physicians practicing outside of academic hospital settings and older physicians are less comfortable prescribing clozapine and are thus appreciative of the safeguards provided by the clozapine REMS program. A negative perception of the clozapine REMS program was also associated with race, with White physicians more likely to report dissatisfaction than American Indian or Alaska Native, Black, Native Hawaiian or Other Pacific Islander physicians. This difference might be due in part to White physicians being more likely to practice in higher socioeconomic settings,^19^ in which REMS program requirements might be perceived as unnecessary. Additional research is needed to examine these hypotheses.

Most physicians surveyed also agreed that the blood testing requirement often caused a delay in patients receiving their medication. The ramifications of such interruptions in treatment can be stark. Discontinuation of clozapine among responders is associated with relapse, hospitalization, and persistent psychosis.^20,21^

Owing to awareness of potential harms in interruptions in treatment from the rollout of the modified clozapine REMS program that was launched on November 15, 2021, the FDA has taken several steps to protect patient and public health. In November 2021, the FDA announced that it would not object to pharmacists dispensing clozapine without REMS authorization or to wholesalers shipping clozapine to pharmacies and other health care settings without confirming enrollment in the REMS.^17^ In March 2023, the FDA opened a public docket requesting feedback on factors the agency should consider when reviewing proposed changes to REMS administrators.^22^ In September 2023, the FDA announced it would conduct a full reevaluation of the clozapine REMS program.^23^

In considering possible reforms to create the optimal balance of safety and access, a global comparison of policies governing clozapine use would be instructive. In contrast to many other countries, the US permits nonpsychiatrist prescribing and retail dispensing of clozapine. Several countries had a shorter duration of weekly testing and ended monitoring requirements after the first few months.^24^ Long-term outcomes of clozapine initiators in these countries should be compared.

### Limitations

This study has limitations. As with all surveys, our study was subject to participation, recall, and response biases. Specifically, it is possible that physicians who did not feel strongly about the clozapine REMS program were less likely to participate, that respondents’ memory of the certification process was poor, and that respondents felt pressured to respond in certain ways. We sought to reduce this bias by inviting a random sample of physician prescribers, limiting participation to physicians who had prescribed clozapine in 2021 or 2022, and ensuring the confidentiality of responses. Responses were also affected by the COVID-19 public health emergency, during which time the FDA exercised enforcement discretion as to REMS-required testing.^17^ Additionally, the views of nonprescribers were not included, which may have overestimated the positive perceptions of the program reported in our survey because some physicians with highly negative perceptions may avoid prescribing the drug altogether.

## Conclusions

This survey study of physician prescribers of clozapine offers insight into physician perceptions of and experiences with the clozapine REMS program. In general, most survey respondents were satisfied with the clozapine REMS program, highlighting the clarity of the testing information and usefulness of the drug information disseminated as part of the certification process as well as the patient-physician discussion facilitated by the safe use requirements. Concerns from survey responses included the adequacy of the scope of content in the education materials used in the certification process and delays in patient receipt of medication, in part owing to the burden of required laboratory testing. These issues warrant consideration as part of the ongoing FDA review of the clozapine REMS program.

## Notes

Supplement 1. **eAppendix.** Survey Instrument **eFigure 1.** Responses to Survey Questions Related to the Risk Evaluation and Mitigation Strategy (REMS) Certification Process **eFigure 2.** Responses to Survey Questions Related to the Risk Evaluation and Mitigation Strategy (REMS) Safe Use Requirements

Supplement 2.
Data Sharing Statement
