# Supplementary material for: Physician Experiences With and Perspectives on Clozapine Prescribing
Source: JAMA Netw Open. 2025 Feb 13;8(2):e2459311. doi: 10.1001/jamanetworkopen.2024.59311 (PMC11826364; doi:10.1001/jamanetworkopen.2024.59311)
Supplement: Supplement 1. — eAppendix. Survey Instrument eFigure 1. Responses to Survey Questions Related to the Risk Evaluation and Mitigation Strategy (REMS) Certification Process eFigure 2. Responses to Survey Questions Related to the Risk Evaluation and Mitigation Strategy (REMS) Safe Use Requirements [file jamanetwopen-e2459311-s001.pdf]

Supplementary Online Content

Sarpatwari A, Lu Z, Russo M, et al. Physician experiences with and perspectives on clozapine prescribing. *JAMA Netw Open*. 2025;8(2):e2459311. doi:10.1001/jamanetworkopen.2024.59311

eAppendix. Survey Instrument

eFigure 1. Responses to Survey Questions Related to the Risk Evaluation and Mitigation Strategy (REMS) Certification Process

eFigure 2. Responses to Survey Questions Related to the Risk Evaluation and Mitigation Strategy (REMS) Safe Use Requirements

This supplementary material has been provided by the authors to give readers additional information about their work.

© 2025 Sarpatwari A et al. *JAMA Network Open*.

Risk Evaluation and Mitigation Strategy (REMS) Programs to Promote Appropriate Medication Use and Knowledge: Physician Surveys on Experiences with REMS Programs

OMB Control Number: 0910-0847
Expiration Date: 12/31/2022

eAppendix. Survey Instrument

Risk Evaluation and Mitigation Strategy (REMS) Programs to Promote Appropriate Medication Use and Knowledge: Physician Surveys on Experiences with REMS Programs

OMB Control Number: 0910-0847
Expiration Date: 12/31/2022

Paperwork Reduction Act Statement: According to the Paperwork Reduction Act of 1995, an agency may not conduct or sponsor, and a person is not required to respond to a collection of information unless it displays a valid OMB control number. The valid OMB control number for this information collection is 0910-0847. The time required to complete this portion of the information collection is estimated to average 2 minutes per response, including the time for reviewing instructions, searching existing data sources, gathering and maintaining the data needed, and completing and reviewing the collection of information.

Send comments regarding this burden estimate or any other aspects of this collection of information, including suggestions for reducing burden to PRAStaff@fda.hhs.gov.

Physician Survey on Clozapine Prescribing

Thank you for agreeing to participate in this survey relating to your experiences prescribing clozapine. This research is being conducted by investigators at Brigham and Women's Hospital / Harvard Medical School on behalf of the US Food and Drug Administration (FDA). If you have NOT prescribed clozapine in the last year, **please write "Have not prescribed clozapine" on the cover of your questionnaire and mail it in the envelope provided in the packet you received in the mail.**

Your participation in the survey is voluntary, and you may withdraw at any time. Your responses will be aggregated with other responses and analyzed in a de-identified manner. The survey methods have been approved by the Institutional Review Board at Brigham and Women's Hospital and the FDA Research Involving Human Subjects Committee.

The survey should take approximately 20 minutes to complete. In addition to the $20 enclosed in this packet, following completion, you will be asked for your email address and emailed an $80 Amazon gift card as a token of appreciation. This survey is not connected in any way with a pharmaceutical manufacturer.

We appreciate your contribution to this important topic. Thank you in advance for your participation!

Instructions for Completing the Survey

- **As a reminder, you can take the survey online if you prefer using the following link and ID:**
  http://REMS.survey-source.com
  UNIQUE ID: [INSERT ID]
- Using a blue or black pen, place an "X" in the box next to the appropriate response as shown: ☒.
- If asked to provide a written response to a question, please PRINT legibly in the space provided.
- If completing the paper questionnaire, please return it in the enclosed postage-paid envelope.

Section A: Prescribing and Certification Requirements

We will start the survey by getting a better understanding of your experience with clozapine.

A1. Approximately when was the last time you prescribed clozapine? ***Please enter month with 2 digits, e.g., 04.***

☐☐ month ☐☐☐☐ year

A2. Approximately how many of your patients have you prescribed clozapine to over the last 3 years?

- ☐1 1-10 patients
- ☐2 11-20 patients
- ☐3 21 or more patients

A3. Approximately how many <u>women of reproductive potential</u> have you prescribed clozapine to over the last 3 years?

- ☐1 None
- ☐2 1-5 patients
- ☐3 6-10 patients
- ☐4 11 or more patients

As you may know, clozapine is subject to a special safety program. Before prescribing clozapine, physicians must go through a certification process administered by the manufacturer. The certification process typically involves such activities as reviewing certain materials, training, and filling out forms.

A4. Approximately how many years ago did you <u>first</u> complete the certification process for clozapine? ***If less than a year ago, please enter "0."***

☐☐ years ago

A5. How well do you recall the certification process that allowed you to begin to prescribe clozapine?

- ☐1 Very well
- ☐2 Moderately well
- ☐3 Slightly well
- ☐4 Not well at all

A6. Did the <u>certification process</u> for clozapine provide information on the following risks?

	Yes	No	I don't remember
a. Birth defects (among women of reproductive potential)	☐1	☐2	☐3
b. Decreased hemoglobin count	☐1	☐2	☐3
c. Orthostatic hypotension, bradycardia, and syncope	☐1	☐2	☐3
d. Pulmonary embolism	☐1	☐2	☐3
e. Seizure	☐1	☐2	☐3
f. Severe neutropenia	☐1	☐2	☐3

A7. When you start a patient on clozapine, how often do you discuss the following risks?

	Never (0% of the time)	Rarely (1%-5% of the time)	Sometimes (6%-25% of the time)	Often (26%-50% of the time)	Most of the time (51%-75% of the time)	Always/almost always (76% of the time or more)
a. Birth defects (among women of reproductive potential)	☐1	☐2	☐3	☐4	☐5	☐6
b. Decreased hemoglobin count	☐1	☐2	☐3	☐4	☐5	☐6
c. Orthostatic hypotension, bradycardia, and syncope	☐1	☐2	☐3	☐4	☐5	☐6
d. Pulmonary embolism	☐1	☐2	☐3	☐4	☐5	☐6
e. Seizure	☐1	☐2	☐3	☐4	☐5	☐6
f. Severe neutropenia	☐1	☐2	☐3	☐4	☐5	☐6

© 2025 Sarpatwari A et al. *JAMA Network Open*.

A8. Using a scale from 1 (most) to 4 (least), please rank the following risks to patients receiving clozapine in order of their magnitude of concern to you.

1 Orthostatic hypotension, bradycardia, and syncope ☐
2 Pulmonary embolism ☐
3 Seizure ☐
4 Severe neutropenia ☐

A9. Using a scale from 1 (most) to 5 (least), please rank the usefulness of the following sources of information in contributing to your understanding of the risks of clozapine.

1 Clinical decision support tools (e.g., UpToDate, MicroMedex, ePocrates) ☐
2 Manufacturer sales representatives' presentations or materials ☐
3 Professional colleagues ☐
4 Studies and other articles published in medical journals ☐
5 The drug's FDA-approved labeling ☐

A10. Please indicate to what extent you agree or disagree with the following statements.

	Strongly agree	Somewhat agree	Neither agree nor disagree	Somewhat disagree	Strongly disagree
a. It is reasonable that clozapine has a certification process, while other drugs I prescribe for my patients with schizophrenia, such as olanzapine, do not have a certification process.	☐1	☐2	☐3	☐4	☐5
b. The certification process provided me with useful information about clozapine.	☐1	☐2	☐3	☐4	☐5
c. The certification process for clozapine took too long to complete.	☐1	☐2	☐3	☐4	☐5
d. The educational materials provided as part of the certification process should include information about any clinically important risk of clozapine.	☐1	☐2	☐3	☐4	☐5
e. The educational materials provided as part of the certification process should include information about how well clozapine is expected to work.	☐1	☐2	☐3	☐4	☐5
f. The certification process effectively explained the testing required of patients receiving clozapine.	☐1	☐2	☐3	☐4	☐5
g. Prescribers should be required to pass a quiz covering drug risks and testing requirements to complete the clozapine certification process.	☐1	☐2	☐3	☐4	☐5
h. Physicians should be required to repeat the certification process each year while they are active prescribers of clozapine.	☐1	☐2	☐3	☐4	☐5
i. Physicians should be compensated for having to complete the certification process for clozapine.	☐1	☐2	☐3	☐4	☐5

© 2025 Sarpatwari A et al. *JAMA Network Open*.

Section B: Patient Initiation and Monitoring

As you may know, prior to and while taking clozapine, patients are also required to follow certain "safe use requirements".

B1. At first, how frequently must testing for clozapine under the safe use requirements be performed? *If fewer than 10 weeks, please enter as 2 digits, e.g., 04*.

Every ☐☐ weeks

B2. When you prescribe clozapine, how long, on average, do you or someone on your team spend explaining to patients the safe use requirements related to the drug?

☐1 We do not discuss safe use requirements with my patients.
☐2 5 minutes or less
☐3 6-10 minutes
☐4 11-15 minutes
☐5 More than 15 minutes

B3. Who on your clinical team is <u>primarily</u> responsible for helping patients complete administrative paperwork or enrollment forms involved with the safe use requirements? *Please select only one.*

☐1 I am
☐2 A nurse practitioner or registered nurse
☐3 A physician assistant
☐4 Other (Please specify: ___________________________________)
☐5 No one

B4. Do your patients receive from you or your team any other materials describing the risks of taking clozapine?

☐1 Yes → **ANSWER B5.**
☐2 No → **SKIP TO B6.**

B5. What materials do you or your team provide describing the risks or harms of clozapine? *Please select all that apply.*

☐1 Published articles or stories
☐2 Links to manufacturer website
☐3 Links to any non-manufacturer websites
☐4 Pamphlets or brochures produced by the manufacturer
☐5 Pamphlets or brochures produced by you or your institution
☐6 Other materials (Please specify: ___________________________________)

B6. After learning about the safe use requirements for clozapine, how often do your patients seek another treatment option instead?

☐1 Never (0% of the time)
☐2 Rarely (1%-5% of the time)
☐3 Sometimes (6%-25% of the time)
☐4 Often (26%-50% of the time)
☐5 Most of the time (51%-75% of the time)
☐6 Always/almost always (76% of the time or more)

© 2025 Sarpatwari A et al. *JAMA Network Open*.

B7. In your estimation, how frequently do your patients follow the testing schedule that is part of the safe use requirements?

- ☐1 Never (0% of the time)
- ☐2 Rarely (1%-5% of the time)
- ☐3 Sometimes (6%-25% of the time)
- ☐4 Often (26%-50% of the time)
- ☐5 Most of the time (51%-75% of the time)
- ☐6 Always/almost always (76% of the time or more)

B8. Please indicate to what extent you agree or disagree with the following statements.

	Strongly agree	Somewhat agree	Neither agree nor disagree	Somewhat disagree	Strongly disagree
a. Testing under the safe use requirements is clinically necessary.	☐1	☐2	☐3	☐4	☐5
b. The paperwork involved with the safe use requirements facilitates discussion about clozapine between patients and me or my team.	☐1	☐2	☐3	☐4	☐5
c. The safe use requirements are burdensome for most patients.	☐1	☐2	☐3	☐4	☐5
d. The safe use requirements have often caused a delay in my patients receiving their medication.	☐1	☐2	☐3	☐4	☐5
e. Insurance issues have often caused a delay in my patients receiving their medication.	☐1	☐2	☐3	☐4	☐5
f. Insurance issues are more burdensome than safe use requirements for most patients.	☐1	☐2	☐3	☐4	☐5

Section C: Overall Experiences and Perceptions and Reforms

C1. Please rate how easy or hard it is to complete the following tasks related to prescribing clozapine.

	Very easy	Somewhat easy	Neither easy nor hard	Somewhat hard	Very hard
a. The physician certification process	☐1	☐2	☐3	☐4	☐5
b. The patient enrollment process	☐1	☐2	☐3	☐4	☐5
c. Testing patients	☐1	☐2	☐3	☐4	☐5
d. Reporting testing findings	☐1	☐2	☐3	☐4	☐5

C2. How willing would you be to prescribe clozapine if it was <u>not</u> subject to…?

	Very willing	Somewhat willing	Neither willing nor unwilling	Somewhat unwilling	Very unwilling
a. Physician certification requirements	☐1	☐2	☐3	☐4	☐5
b. Patient safe use requirements	☐1	☐2	☐3	☐4	☐5

C3. How frequently are patients needing clozapine referred to you by other physicians in your specialty because they are not certified to prescribe it?

- ☐1 Often
- ☐2 Sometimes

© 2025 Sarpatwari A et al. *JAMA Network Open*.

☐3 Never

C4. Please indicate to what extent you agree or disagree with the following statements.

Overall, the positives of the…	Strongly agree	Somewhat agree	Neither agree nor disagree	Somewhat disagree	Strongly disagree
a. Prescriber certification process for clozapine outweighs the negatives.	☐1	☐2	☐3	☐4	☐5
b. Patient safe use requirements for clozapine outweigh the negatives.	☐1	☐2	☐3	☐4	☐5

C5. What feedback would you give the FDA or manufacturer on the <u>physician certification process</u> for clozapine?
Please print clearly in the box below. If you need more space, continue on the back cover. Be sure to include the question number.

C6. What feedback would you give the FDA or manufacturer on the <u>patient safe use requirements</u> for clozapine?
Please print clearly in the box below. If you need more space, continue on the back cover. Be sure to include the question number.

Section D: Pandemic Impact

D1. Did you prescribe clozapine prior to the start of the COVID-19 pandemic in March 2020?

☐1 Yes → **ANSWER D2.**
☐2 No → **SKIP TO D3.**

D2. Please rate how much easier or harder it was to complete the following tasks related to prescribing clozapine during vs. before the pandemic.

	Much easier during the pandemic	Somewhat easier during the pandemic	Neither easier nor harder	Somewhat harder during the pandemic	Much harder during the pandemic
a. The patient enrollment process	☐1	☐2	☐3	☐4	☐5
b. Testing patients	☐1	☐2	☐3	☐4	☐5
c. Reporting testing findings	☐1	☐2	☐3	☐4	☐5

© 2025 Sarpatwari A et al. *JAMA Network Open*.

D3. **In March 2020, the FDA announced it would permit drug manufacturers and health care providers to make accommodations for laboratory tests required under the drug safety programs during the COVID-19 pandemic, such as allowing patients to take blood tests less frequently.**

Were you aware of this policy?

☐1 Yes
☐2 No

D4. **Did the <u>manufacturers</u> of clozapine change the drug's <u>blood testing requirements</u> in response to the pandemic?**

☐1 Yes
☐2 No
☐3 I don't know

D5. **Did <u>you</u> change <u>blood testing requirements</u> for your patients taking clozapine in response to the pandemic (independent of the drug manufacturers)?**

☐1 Yes (Describe briefly: ____)
☐2 No

Section E: Demographics

E1. **Which of the following best describes your specialty? *You may select up to 2.***

☐1 Allergy/Immunology
☐2 Anesthesiology
☐3 Cardiology
☐4 Dermatology
☐5 Endocrinology
☐6 Emergency Medicine
☐7 Family/General Practice
☐8 Geriatrics
☐9 Internal Medicine
☐10 Medical Genetics
☐11 Neurological Surgery
☐12 Nephrology
☐13 Neurology
☐14 Obstetrics/Gynecology
☐15 Oncology
☐16 Ophthalmology
☐17 Orthopedics
☐18 Otolaryngology
☐19 Pathology
☐20 Pediatrics
☐21 Physical Medicine and Rehab
☐22 Plastic Surgery
☐23 Preventive Medicine
☐24 Psychology
☐25 Pulmonology
☐26 Radiology
☐27 Rheumatology
☐28 Sleep medicine
☐29 Surgery
☐30 Urology
☐31 Other (Please specify: ____________________)

© 2025 Sarpatwari A et al. *JAMA Network Open*.

E2. In what ZIP code is your practice located?

☐☐☐☐☐

E3. In what clinical settings do you prescribe clozapine? ***You may select more than one.***

☐1 Outpatient clinic (solo practice)
☐2 Outpatient clinic (group practice)
☐3 Community hospital (non-military/VA)
☐4 Academic hospital (non-military/VA)
☐5 Military or VA hospital
☐6 Other (Please specify: ________________________________)

[UNIQUE ID]-

© 2025 Sarpatwari A et al. *JAMA Network Open*.

eFigure 1. Responses to Survey Questions Related to the Risk Evaluation and Mitigation Strategy (REMS) Certification Process

Percentages are based on the number of responses to the question.

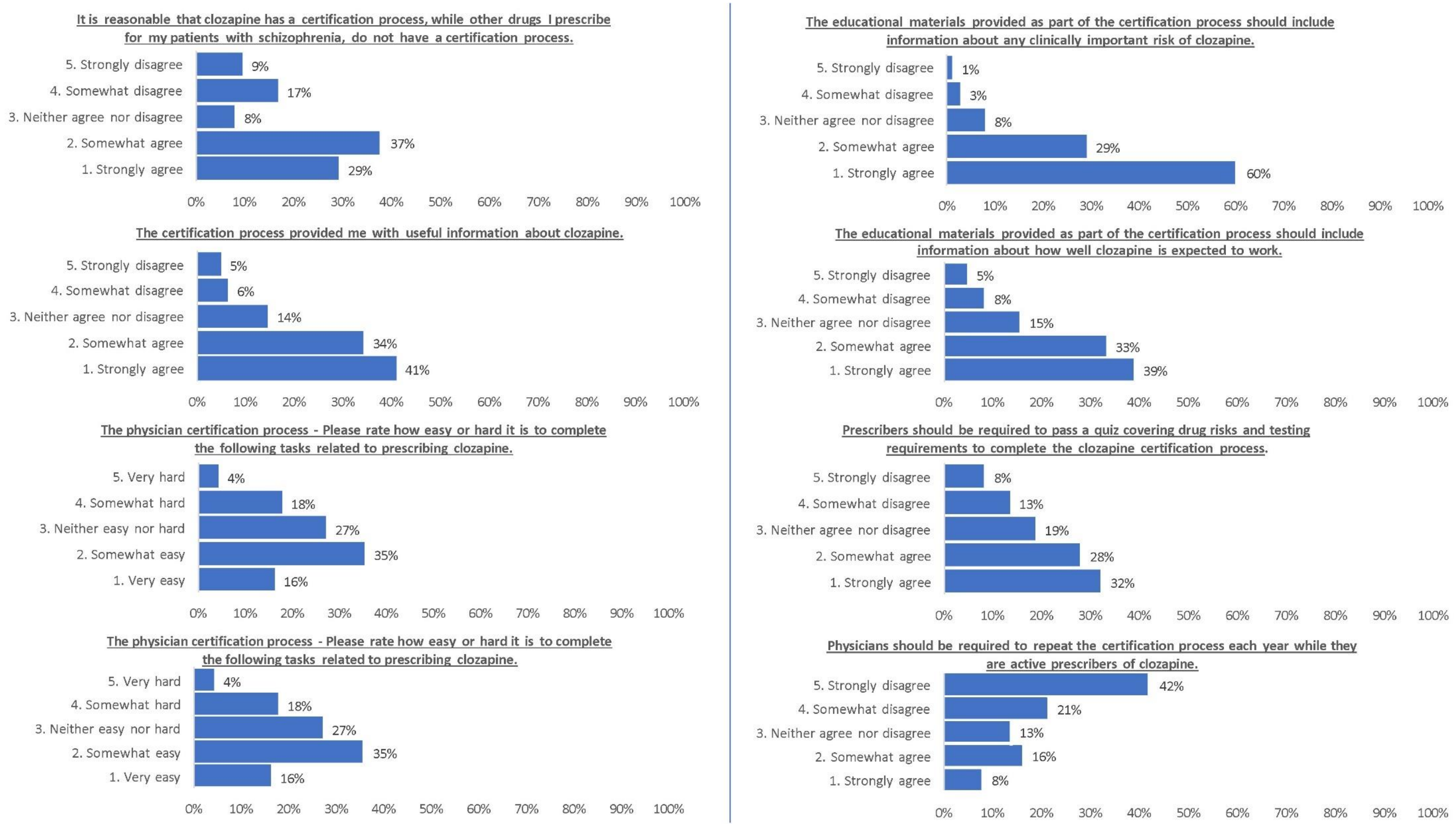

© 2025 Sarpatwari A et al. *JAMA Network Open*.

eFigure 2. Responses to Survey Questions Related to the Risk Evaluation and Mitigation Strategy (REMS) Safe Use Requirements

Percentages are based on the number of responses to the question.

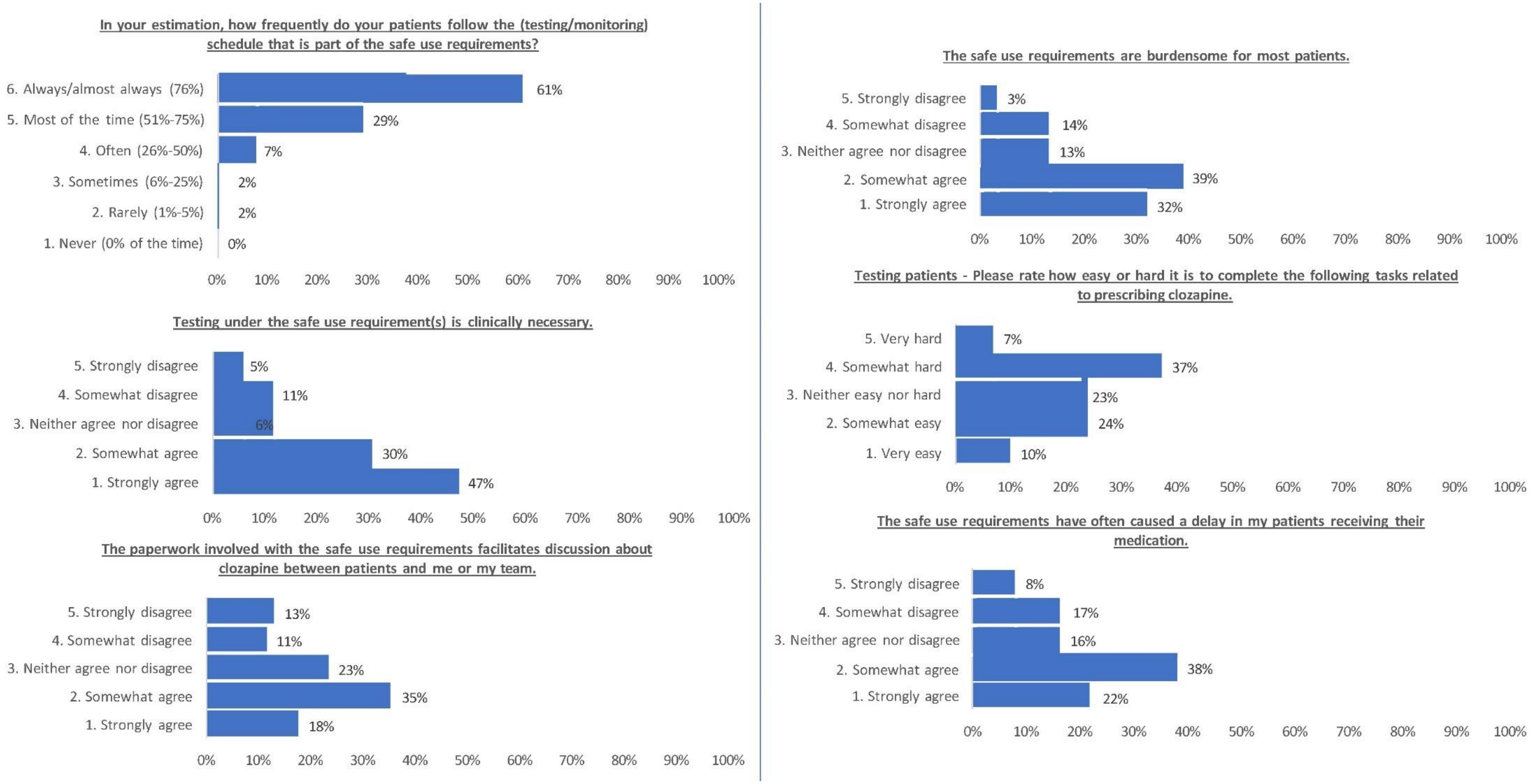

© 2025 Sarpatwari A et al. *JAMA Network Open*.
