# Supplementary material for: Physician Experiences With and Perspectives on Clozapine Prescribing
Source: JAMA Netw Open. 2025 Feb 13;8(2):e2459311. doi: 10.1001/jamanetworkopen.2024.59311 (PMC11826364; doi:10.1001/jamanetworkopen.2024.59311)
Supplement: Supplement 2. — Data Sharing Statement [file jamanetwopen-e2459311-s002.pdf]

Data Sharing Statement

Sarpatwari. Physician Experiences With and Perspectives on Clozapine Prescribing. *JAMA Netw Open*. Published February 13, 2025. doi:10.1001/jamanetworkopen.2024.59311

Data
Data available: Yes
Data types: Data dictionary, Deidentified participant data
How to access data: Requests must be sent to the corresponding author
When available: With publication

Supporting Documents
Document types: None

Additional Information
Who can access the data: Researchers whose proposed use of the data has been approved
Types of analyses: For research purposes
Mechanisms of data availability: With investigator support
